# Damage control strategy in peripheral vascular injury caused by firearm: A case report

**DOI:** 10.3892/mi.2025.238

**Published:** 2025-05-05

**Authors:** Selene Magdalena Preciado Sepúlveda, Elba Alejandra Muñoz Rolón, Florencia Seimandi Soto

**Affiliations:** Division of General Surgery, Hospital Regional Universitario, 28010 Colima, Colima, Mexico

**Keywords:** peripheral vascular injury, limb ischemia, damage control

## Abstract

In peripheral vascular injury, the need for amputation following prolonged ischemia can be avoided through rapid revascularization. However, critically ill patients often cannot undergo single-stage surgery; thus, the use of vascular shunts temporarily alleviates ischemia, protecting the limb until definitive treatment. The present study describes the case of a 28-year-old male patient with a vascular injury in the right leg secondary to a firearm wound. Following admission, he developed grade IV hypovolemic shock. The patient underwent surgery, where a complete transection of the popliteal artery was found, and a shunt was placed for ischemic control. During surgery, he developed metabolic acidosis and was transferred to the intensive care unit for stabilization. The patient was transfused and underwent a second procedure for an inverted saphenous vein graft. The patient exhibited good clinical progression with palpable distal pulses in the affected limb. The damage control strategy in the case described herein allowed for the preservation of the affected limb, with no complications or need for further surgical intervention observed.

## Introduction

Peripheral vascular injury is a severe although infrequent complication of acute trauma (1). Its incidence has increased due to the rise in workplace accidents, traffic incidents and firearm assaults (2). Regardless of its etiology, the vessels in the lower limbs are usually the most affected (3), particularly the femoral artery and vein (4). Although the mortality rate for these injuries does not exceed 10% (5), the potential functional repercussions can be incapacitating (6), both due to neurological sequelae and in cases of limb loss through amputation (7). Although historically, the latter has been the most effective treatment for the survival of patients (8), since the late 20th century, the therapeutic approach of ‘damage control’ has been adopted, aiming to preserve both life and limb functionality (9). Prolonged ischemia is the main predictor for amputation (10), this approach is indicated in up to 85% of cases where ischemia lasts longer than six hours (11). Therefore, ensuring rapid reperfusion is an imperative goal in the treatment of vascular injury (12). This can be achieved through the placement of stents, bypasses (13) or venous grafts during the intraoperative period (14). However, on some occasions, the severity of the injury requires multidisciplinary management causing delays in revascularization (15). Providing temporary relief from ischemia through shunts helps maintain tissue perfusion until the patient stabilizes (16), at which point definitive treatment can proceed (17). This approach improves the overall prognosis of critically ill patients or those with complex vascular trauma (18), reducing mortality while preserving the limb (19,20). The present study describes the case of a patient with severe vascular injury, who despite an unstable hemodynamic state, was successfully treated using this damage control strategy.

## Case report

A 28-year-old male with no significant medical history, was admitted to the Emergency Department of Hospital Regional Universitario (Colima, Mexico) due to a hemorrhagic injury in the right lower limb secondary to a firearm wound, with a progression time of 1 h and 20 min. In the initial assessment, the patient was found to be hemodynamically unstable, presenting with grade IV hypovolemic shock, with an entry wound on the anteromedial side of the right leg and an exit wound on the lateral side. A pulsatile and expanding hematoma was observed in the area, along with a gaping wound with musculoskeletal tissue loss secondary to short blunt trauma. Absent pulses were noted in the dorsalis pedis, posterior tibial and anterior tibial arteries; the affected limb was pale and poikilothermic, with a capillary refill time of 6 sec. Sensitivity and muscle strength were preserved and the revised trauma score (RTS) was calculated at 7.8408. Management was initiated with oxygen therapy, hyperhydration, the transfusion of blood products, as well as antibiotics (cclindamycin: 600 mg, i.v. administration, every 8 h; and cefixime: 2 g i.v. administration, every 12 h) and analgesia (buprenorphine: 0.3 mg i.v. administration, every 8 h, combined with the continuous intravenous infusion of paracetamol at a dose of 1 g). Following the aforementioned, the decision was made to take the patient urgently to the operating room for vascular exploration.

### Treatment technique

**Figure 1:**
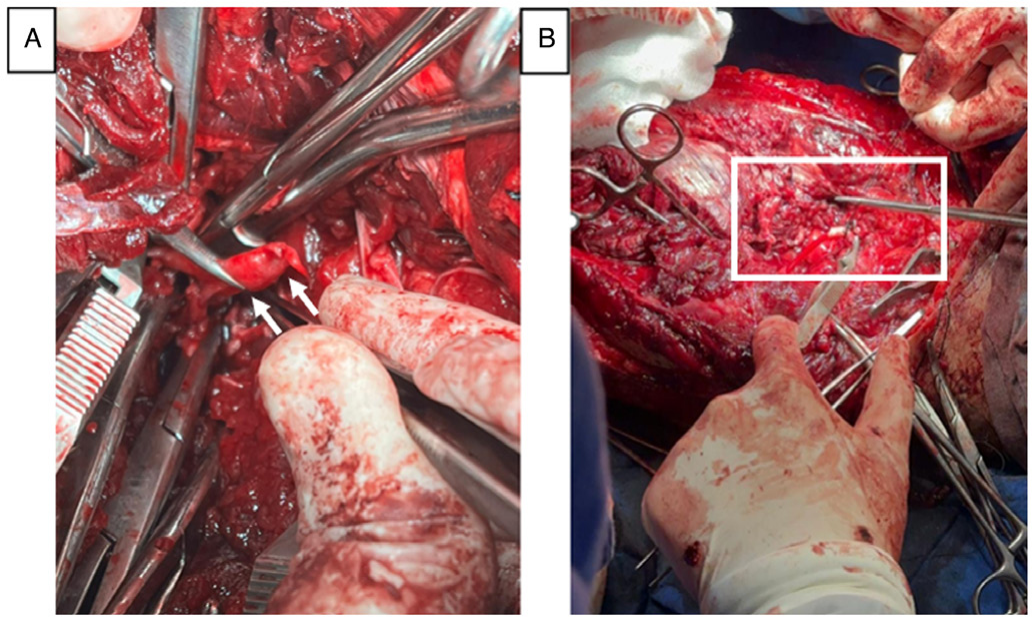
Surgical approach during the initial procedure for vascular control. Surgical approach during the initial procedure for vascular control. Image captured during the first surgery, showing multiple hemostatic clamps used to control bleeding from the injured artery (center-upper left of the image). (A) Arrows indicate the sites of hemostatic control on the popliteal artery. (B) Intraoperative image, with the black square box highlighting the location of the arterial shunt placement.

Under general anesthesia, the patient was positioned in a prone position, and a posterior surgical approach was made to the left popliteal artery. Distal vascular control of the popliteal artery and vein was achieved. Subsequently, the incision was extended proximally to explore the hematoma in the posterior region of the right leg, thus opening the superficial and deep posterior compartments, revealing a complete transection of the popliteal artery with a discontinuity of ~5 cm, as well as of the popliteal vein and the deep peroneal nerve, with no possibility of primary anastomosis. Sodium heparin was administered at a dose of 100 IU/kg. Given the hemodynamic condition of the patient and the development of intraoperative acidosis, a damage control strategy was implemented, with a popliteal-popliteal shunt using an 8 French feeding tube and the ligation of the thrombosed popliteal vein (Fig. 1). The proximal clamp was removed, revealing the presence of arterial flow and distal pulses, though diminished in amplitude, with a capillary refill time of 4 sec. The surgical wound was cleaned, and partial closure was performed. The total blood loss during the surgery and emergency room stay was 4,000 ml. The patient received one red blood cell concentrate during his stay in the emergency room and two in the operating room along with one unit of fresh-frozen plasma. The patient was admitted to the intensive care unit for comprehensive support and to improve hemodynamic conditions for a second definitive surgical procedure.

**Figure 2:**
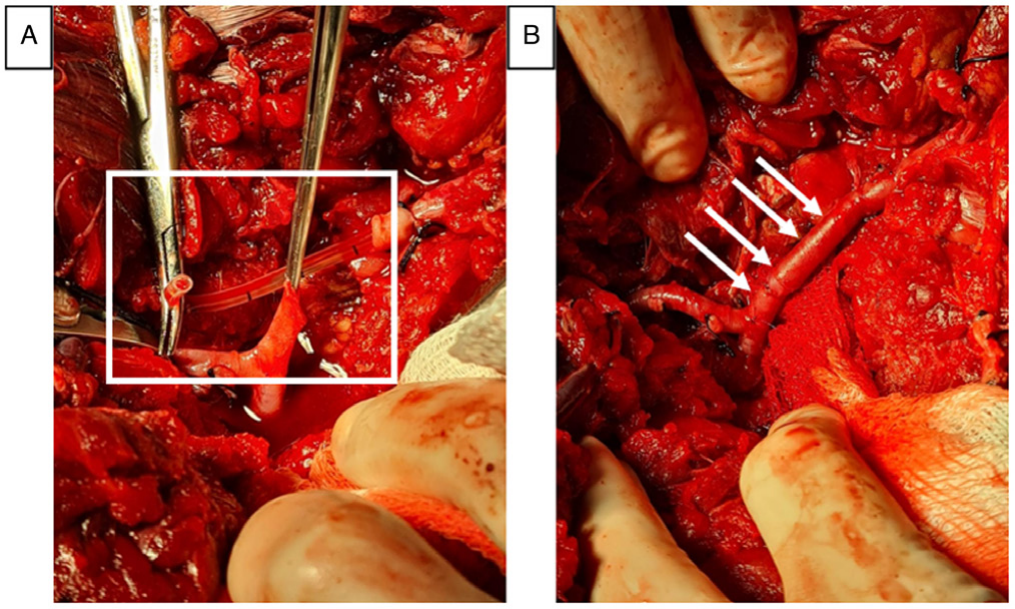
Surgical approach during the second intervention. Surgical approach during the second intervention. The image captured was during the final surgery, illustrating the bifurcation of the severed artery where the vascular anastomosis was performed. (A) The black square box displays the arterial shunt along with the feeding catheter after removal. (B) Arrows indicate the performed bypass, with the arrow tips highlighting the pinpoint lesion in the anterior tibial artery at the popliteal artery bifurcation.

After 10 h, the patient regained hemodynamic stability; however, signs of acute ischemia began to appear, prompting a reintervention for limb revascularization. For this, the contralateral greater saphenous vein was harvested for use as an autologous graft. Upon the reopening of the previous incision, the thrombosed shunt was found, which was dismantled. Additionally, a previously unnoticed punctate lesion of 1 mm in diameter was discovered in the anterior tibial artery, which was repaired with a simple suture using 7-0 vascular Prolene. A thrombectomy was performed proximally and distally with a 3 French Fogarty catheter, retrieving a fresh thrombus. Subsequently an interposition graft was made in the popliteal artery using an inverted saphenous vein, with end-to-end anastomosis using 6-0 vascular Prolene. Upon removing the proximal clamp, adequate arterial flow and the presence of distal pulses were observed. Posterior and lateral fasciotomies were performed to prevent compartment syndrome, with partial closure of the surgical wound, and no drains were placed. Sodium heparin was administered at a dose of 100 IU/kg (Fig. 2).

The patient was stable in the immediate post-operative period. No signs of compartment syndrome or reperfusion syndrome were observed. However, follow-up laboratory tests revealed normal renal function, with a serum creatinine level of 1.0 mg/dl, a urea level of 30 mg/dl, blood urea nitrogen (BUN) level of 15 mg/dl, and an estimated glomerular filtration rate (eGFR) >90 ml/min/1.73 m². Serum electrolytes were within normal limits, including sodium at 140 mmol/l, potassium at 4.2 mmol/l, chloride at 102 mmol/l, bicarbonate at 24 mmol/l, total calcium at 9.5 mg/dl, magnesium at 2.0 mg/dl and phosphorus at 3.5 mg/dl. Cardiac biomarkers were also within the expected range, with a total creatine phosphokinase level of 100 U/l, a CPK-MB fraction of 20 U/l, a troponin I level of 0.02 ng/ml, a troponin T level of 0.005 ng/ml, a myoglobin concentration of 50 ng/ml, and a lactate dehydrogenase level of 180 U/l. Complete blood count analysis revealed severe anemia with a hemoglobin concentration of 5 g/ds, without additional abnormalities in the leukocyte or platelet counts. Arterial blood gas analysis demonstrated metabolic acidosis without other significant alterations. Therefore, medical management with fluid therapy, diuretics and the close monitoring of renal function was initiated. Subsequently, the patient had an adequate clinical evolution, with resolution of the rhabdomyolysis symptoms. He was discharged 10 days following the second surgical procedure, exhibiting adequate distal pulses and with no signs of infection or other early complications. As regards follow-up, the patient was evaluated at 1 and 3 months after the intervention. At that time, adequate vascularization was confirmed through the palpation of distal pulses and the full functionality of the salvaged limb, along with proper wound healing. No ultrasound images were available for this timeframe; however, based on clinical observations, the functionality of the limb was maintained.

## Discussion

The present study described the case of a young male patient with a firearm-induced vascular injury in the lower limb. The complication resulted in grade IV hypovolemic shock and metabolic acidosis, necessitating urgent intervention for hemostatic control. However, due to the complexity of the injuries and the presence of ischemia, a damage control strategy was implemented, leading to limb preservation and a favorable postoperative recovery following the definitive intervention.

The case described herein highlights the complexity of limb salvage decisions in acute vascular injuries, where the option between amputation and preservation depends on multiple factors, including the severity of ischemia and the patient's overall condition (21). The patient had no significant medical history, and no fractures were identified. Despite this, the development of grade IV hypovolemic shock and metabolic acidosis posed challenges in patient management, as these conditions require immediate surgical intervention in such injuries (22).

Although simple indices, such as the mangled extremity score (MES) or the mangled extremity severity index (MESI), can help determine the most appropriate treatment for acute vascular injuries (23), functional and individual patient factors should also be considered. While survival rates following amputation can reach 100%, depending on the technique used and the condition of the patient (24), the long-term consequences are critical. Studies have reported substantial psycho-emotional distress, increased mobility-related costs and disruptions in interpersonal relationships, particularly when amputation results from traumatic events (25), as in the case described herein. Additionally, phantom limb pain, somatosensory alterations (26) and weight gain (27) are commonly reported following amputation. These negative effects can be mitigated through early rehabilitation, physiotherapy, and prosthesis adaptation (28). However, previous research has indicated that while cerebral cortex reorganization occurs following upper limb amputations, it is less effective in lower limb amputations. Consequently, symptoms such as phantom limb pain, reduced dexterity and impaired balance tend to be more persistent (14).

Conversely, limb salvage reduces the risk of long-term complications, such as cardiovascular diseases and arterial disease (29). Although not always possible, the use of tools such as vascular shunts can extend the time window for intervention (30). Additionally, the use of grafts as in the case in the present study, both anatomical and extra-anatomical, can improve the condition of the patient until the definitive intervention (31), preserving the affected limb and resulting in lower rates of future reinterventions (32).

In the patient in the present study, factors such as a young age and the absence of comorbidities supported the decision to manage the vascular injury with a damage control approach. This choice was based on the greater capacity of the patient for recovery and tissue regeneration. The literature emphasizes that damage control techniques can be particularly beneficial in such cases, as they allow for the restoration of distal perfusion through temporary vascular shunts prior to definitive revascularization. This is particularly relevant when the duration of ischemia is <6 h (2,33), as observed in the patient in the present study. Indeed, reports of similar cases have described favorable outcomes with the use of damage control strategies in young patients. In a previously documented case, a young male patient sustained severe vascular trauma following a shark attack, resulting in multiple fractures of his right pelvic limb and hemorrhage leading to hypovolemic shock (34). Following initial management, a damage control approach was selected, with intervention on the popliteal artery and vein. Following reconstruction, limb functionality was preserved without neurological or vascular deficits (34). Similarly, another study described the case of a young female patient who sustained a tibia and fibula fracture in her left leg after being struck by a vehicle; in that case, limb preservation was achieved through direct vascular repair of the popliteal vessels (35). Likewise, in another study, a 33-year-old male presented with a Gustilo-Anderson type IIIA fracture of the distal tibia and fibula, accompanied by a closed calcaneus fracture and tibial nerve transection (36). Although amputation was considered, the decision was made to preserve the limb using a nerve allograft. At 29 months of post-operative follow-up, the patient demonstrated satisfactory functional recovery without the need for major revision, grafting, arthrodesis or amputation (36).

Furthermore, in support of the damage control approach, a recent meta-analysis found that patients who underwent limb salvage demonstrated improved psychological well-being compared to those who underwent amputations, despite the possibility of reinterventions and a prolonged recovery period (37). Additionally, the Lower Extremity Assessment Project (LEAP) study revealed that although long-term functional outcomes may be similar between both strategies, limb reconstruction is generally preferred by patients due to the negative psychosocial effects of amputation, which include depressive disorders and anxiety (38).

Moreover, while immediate amputation reduces the risk of developing late complications, such as infections and multiple reinterventions, recent research has highlighted that the use of temporary vascular shunts and delayed revascularization can significantly improve the success rate of limb salvage without compromising patient safety (33). There is evidence to suggest that careful selection of candidates for the damage control strategy can optimize outcomes, avoiding the adverse consequences associated with amputation, such as phantom limb pain and difficulty adapting to prostheses (23).

However, it is important to acknowledge the inherent risks associated with this approach, many of which arise during the procedure itself. Among the most frequent complications are vascular thrombosis, infections and the development of anastomotic stenosis, all of which can jeopardize limb viability (39). Fortunately, in the case described herein, the use of heparin and the close monitoring of the progress of the patient reduced the risk of thrombosis during the intraoperative period until the definitive surgical approach could be implemented. Moreover, the risk of anastomotic stenosis can be mitigated through surgical techniques, such as balloon angioplasty, stent placement, or the use of temporary shunts in selected cases. These shunts help prolong vascular patency (40), which is why these were implemented in the patient in the present study, facilitating stabilization and enabling a subsequent definitive intervention.

In conclusion, based on the evidence presented, the damage control strategy emerges as a highly favorable approach in cases of acute vascular injury, balancing the risks and benefits associated with limb salvage. Finally, it is noteworthy that the patient exhibited satisfactory progress 3 months following the intervention, at which point full limb functionality was restored with no apparent clinical deficits. Despite this positive outcome, future studies should consider longer follow-up periods and imaging evaluations to assess the long-term efficacy of the damage control strategy.

## Data Availability

The data generated in the present study may be requested from the corresponding author.

## References

[1] Hundersmarck D, Hietbrink F, Leenen LPH, De Borst GJ, Heng M (2022). Blunt popliteal artery injury following tibiofemoral trauma: Vessel-first and bone-first strategy. Eur J Trauma Emerg Surg.

[2] Agarwal P, Kukrele R, Sharma D (2022). Delayed revascularization of extremities following vascular injuries: Challenges and outcome. J Orthop.

[3] Herrera MA, Millán M, Del Valle AM, Betancourt-Cajiao M, Caicedo Y, Caicedo I, Gallego LM, Rivera D, Parra MW, Ordoñez CA (2021). Damage control of peripheral vascular trauma-don't be afraid of axillary or popliteal fosses. Colomb Med (Cali).

[4] Himura H, Uchida K, Miyashita M, Mizobata Y (2021). Successful limb salvage beyond the golden time following blunt traumatic open complete transection of the femoral artery and vein in a patient with cardiac arrest: A case report. Surg Case Rep.

[5] Potter HA, Alfson DB, Rowe VL, Wadé NB, Weaver FA, Inaba K, O'Banion LA, Siracuse JJ, Magee GA (2021). Endovascular versus open repair of isolated superficial femoral and popliteal artery injuries. J Vasc Surg.

[6] Priyadarshini P, Kaur S, Gupta K, Kumar A, Alam J, Bagaria D, Choudhary N, Gupta A, Sagar S, Mishra B, Kumar S (2025). Protocolized approach saves the limb in peripheral arterial injury: A decade experience. Chin J Traumatol.

[7] Liu G, Chen J, Xiang Z (2022). Five-year outcomes of trauma-specific function in patients after acute blunt popliteal artery injury: A matched cohort analysis. J Orthop Surg Res.

[8] Melton SM, Croce MA, Patton JH Jr, Pritchard FE, Minard G, Kudsk KA, Fabian TC (1997). Popliteal artery trauma. Systemic anticoagulation and intraoperative thrombolysis improves limb salvage. Ann Surg.

[9] Semaan E, Hamburg N, Nasr W, Shaw P, Eberhardt R, Woodson J, Doros G, Rybin D, Farber A (2010). Endovascular management of the popliteal artery: Comparison of atherectomy and angioplasty. Vasc Endovascular Surg.

[10] Kleinsorge GHD, Teixeira PGR, Pfannes CCB, do Lago RDV, Abib SCV (2022). Prognostic factors in treatment of traumatic femoropopliteal arterial injuries at a Brazilian trauma center. J Vasc Bras.

[11] Lian WS, Das SK, Hu XX, Zhang XJ, Xie XY, Li MQ (2020). Efficacy of intra-arterial catheter-directed thrombolysis for popliteal and infrapopliteal acute limb ischemia. J Vasc Surg.

[12] Dua A, Patel B, Desai SS, Holcomb JB, Wade CE, Coogan S, Fox CJ (2014). Comparison of military and civilian popliteal artery trauma outcomes. J Vasc Surg.

[13] Nguyen A, Tiziano T, Beckermann J, Wildenberg J, Carmody T (2022). Endovascular repair of a traumatic popliteal artery injury. Cureus.

[14] Garge S, Vyas P, Rathod K, Jaggi S, Talwar I (2016). Leaking pseudoaneurysm of lower limb saphenous vein graft: A rare complication and its successful treatment by endovascular embolization. BJR Case Rep.

[15] Edwards J, Treffalls RN, Abdou H, Stonko DP, Walker PF, Morrison JJ (2022). Lower extremity staged revascularization (LESR) as a new innovative concept for lower extremity salvage in acute popliteal artery injuries: A hypothesis. Patient Saf Surg.

[16] Feliciano DV (2020). Long-term intra-arterial shunt. Trauma Surg Acute Care Open.

[17] Golledge J (2022). Surgical revascularization-best for limb ischemia?. N Engl J Med.

[18] Ratnayake AS, Bala M, Fox CJ, Jayatilleke AU, Thalgaspitiya SPB, Worlton TJ (2022). A critical appraisal of impact of compounding factors in limb salvage decision making in combat extremity vascular trauma. BMJ Mil Health.

[19] Urrechaga E, Jabori S, Kang N, Kenel-Pierre S, Lopez A, Rattan R, Rey J, Bornak A (2022). Traumatic lower extremity vascular injuries and limb salvage in a civilian urban trauma center. Ann Vasc Surg.

[20] Bunn C, Kulshrestha S, Di Chiaro B, Maduekwe U, Abdelsattar ZM, Baker MS, Luchette FA, Agnew S (2021). A leg to stand on: Trauma center designation and association with rate of limb salvage in patients suffering severe lower extremity injury. J Am Coll Surg.

[21] Shishehbor MH, White CJ, Gray BH, Menard MT, Lookstein R, Rosenfield K, Jaff MR (2016). Critical limb ischemia: An expert statement. J Am Coll Cardiol.

[22] Fluck F, Augustin AM, Bley T, Kickuth R (2020). Current treatment options in acute limb ischemia. Rofo.

[23] Nayar SK, Alcock HMF, Edwards DS (2022). Primary amputation versus limb salvage in upper limb major trauma: A systematic review. Eur J Orthop Surg Traumatol.

[24] Panhelleux B, Shalhoub J, Silverman AK, McGregor AH (2022). A review of through-knee amputation. Vascular.

[25] Schober TL, Abrahamsen C (2022). Patient perspectives on major lower limb amputation-a qualitative systematic review. Int J Orthop Trauma Nurs.

[26] Littman AJ, Thompson ML, Arterburn DE, Bouldin E, Haselkorn JK, Sangeorzan BJ, Boyko EJ (2015). Lower-limb amputation and body weight changes in men. J Rehabil Res Dev.

[27] AlMehman DA, Faden AS, Aldahlawi BM, Bafail MS, Alkhatieb MT, Kaki AM (2022). Post-amputation pain among lower limb amputees in a tertiary care hospital in Jeddah, Saudi Arabia: A retrospective study. Saudi Med J.

[28] Ülger Ö, Yıldırım Şahan T, Çelik SE (2018). A systematic literature review of physiotherapy and rehabilitation approaches to lower-limb amputation. Physiother Theory Pract.

[29] Zhu Y, Wu X, Zhang W, Zhang H (2023). Limb-salvage surgery versus extremity amputation for early-stage bone cancer in the extremities: A population-based study. Front Surg.

[30] Barros D'Sa AAB, Harkin DW, Blair PHB, Hood JM, McIlrath E (2006). The belfast approach to managing complex lower limb vascular injuries. Eur J Vasc Endovasc Surg.

[31] Di Primio M, Angelopoulos G, Lazareth I, Priollet P, Zins M, Emmerich J, Yannoutsos A (2020). Innovative endovascular approach for limb salvage in critical limb ischemia. J Med Vasc.

[32] Sousa RS, Oliveira-Pinto J, Mansilha A (2020). Endovascular versus open repair for popliteal aneurysm: A review on limb salvage and reintervention rates. Int Angiol.

[33] Qureshi MK, Ghaffar A, Tak S, Khaled A (2020). Limb salvage versus amputation: A review of the current evidence. Cureus.

[34] Khalil A (2021). Patient survival and limb salvage after shark attack with major vascular injury: A case report. Chin J Traumatol.

[35] Giordano V, Souza FS, Belangero WD, Pires RE (2021). Limb salvage after lower-leg fracture and popliteal artery transection-the role of vessel-first strategy and bone fixation using the ilizarov external fixator device: A case report. Medicina (Kaunas).

[36] Mercer DM, Nguyen HM, Curtis W, Heifner JJ, Chafey DH (2023). Consideration for limb salvage in place of amputation in complex tibial fracture with neurovascular injury: A case report. Iowa Orthop J.

[37] Serlis A, Sgardelis P, Vampertzis T, Rizavas K, Poulios P, Konstantopoulos G (2025). Complex limb injuries: Limb salvage versus amputation-a mini review and meta-analysis. Adv Orthop.

[38] Okereke I, Abdelfatah E (2022). Limb salvage versus amputation for the mangled extremity: Factors affecting decision-making and outcomes. Cureus.

[39] Harnarayan P, Islam S, Harnanan D, Bheem V, Budhooram S (2021). Acute upper limb ischemia: Prompt surgery and long-term anticoagulation prevent limb loss and debilitation. Vasc Health Risk Manag.

[40] Mejia D, Warr SP, Delgado-López CA, Salcedo A, Rodríguez-Holguín F, Serna JJ, Caicedo Y, Pino LF, González-Hadad A, Herrera MA (2021). Reinterventions after damage control surgery. Colomb Med (Cali).

